# Myoepithelial Carcinoma of the Breast Treated with Surgery and Chemotherapy

**DOI:** 10.1155/2013/164761

**Published:** 2013-07-30

**Authors:** Yumi Endo, Hiroshi Sugiura, Hiroko Yamashita, Satoru Takahashi, Nobuyasu Yoshimoto, Mai Iwasa, Tomoko Asano, Tatsuya Toyama

**Affiliations:** ^1^Department of Oncology, Immunology and Surgery, Nagoya City University Graduate School of Medical Sciences, 1 Kawasumi, Mizuho-cho, Mizuho-ku, Nagoya 467-8601, Japan; ^2^Department of Experimental Pathology and Tumor Biology, Nagoya City University Graduate School of Medical Sciences, 1 Kawasumi, Mizuho-cho, Mizuho-ku, Nagoya 467-8601, Japan

## Abstract

Myoepithelial carcinoma (malignant myoepithelioma) of the breast is a rare tumor, for which only a limited number of reports have been published. Most of the reports emphasized diagnosis and pathology but not biological behavior and treatment. We report a 61-year-old patient with breast myoepithelial carcinoma who developed locoregional and distant metastases and received many chemotherapy regimens. She presented with an elastic hard mass of the left breast. Breast conserving surgery was performed as part of both diagnosis and treatment. From the results of histological and immunohistochemical examinations, this case was considered to be a myoepithelial carcinoma. Fifteen months after the completion of adjuvant radiotherapy, distant metastasis of the left parasternal lymph node metastasis developed. She was treated by further excision and received a total of four regimens of chemotherapy including a combination of doxorubicin and cyclophosphamide. She received chemotherapy for 20 months after the diagnosis of metastasis.

## 1. Introduction

Myoepithelial cells have the characteristics of both epithelial and smooth muscle cells [1]. Myoepitheliomas are tumors that arise from myoepithelial cells and show both epithelial and smooth muscle cell characteristics but lack ductal differentiation [2]. Tumors arise mainly in the salivary glands but can occur in the skin, soft tissue, retroperitoneum, breast, and lung [3].

Myoepithelial cells are widely present in the breast where they comprise part of the normal microscopic anatomy of lobules and ducts. However, pure myoepithelial neoplasms of the breast are extremely uncommon and reports are limited to case studies [4]. According to the World Health Organization, myoepithelial lesions are composed of a pure or dominant population of myoepithelial cells. Myoepithelial lesions of the breast encompass myoepithelial hyperplasia, collagenous spherulosis, and myoepithelial carcinoma (malignant myoepithelioma). In the latest classification, myoepithelial carcinoma merges in phenotype with metaplastic carcinoma and has a propensity for metastasis [5]. 

Most reports of myoepithelial carcinoma in the current literature are restricted to case reports with emphasis on diagnosis and pathology [6]. Myoepithelial carcinomas are treated mainly by wide surgical excision, lymph node dissection, and adjuvant radiotherapy [3]. Few case reports of breast myoepithelial carcinoma mentioned chemotherapy, and no details were provided regarding drug regimens or clinical outcomes. There are no data available to help define appropriate treatment for this disease. This report describes a patient with breast myoepithelial carcinoma who developed locoregional and distant metastases and received many chemotherapy regimens.

## 2. Case Report

A 61-year-old female presented with an elastic hard mass measuring 2 cm in the upper central area of the left breast. There were no palpable axillary and subclavicular lymph nodes. General physical and systemic examinations were unremarkable. Mammography showed a circumscribed mass in the upper portion of the left breast. On ultrasound examination, a 1.4 cm lobular, partially indistinct marginated and hypoechoic mass was located in the upper inner area of the left breast. Computed tomography (CT) revealed a lobular mass with enhanced margins. 

**Figure 1:**
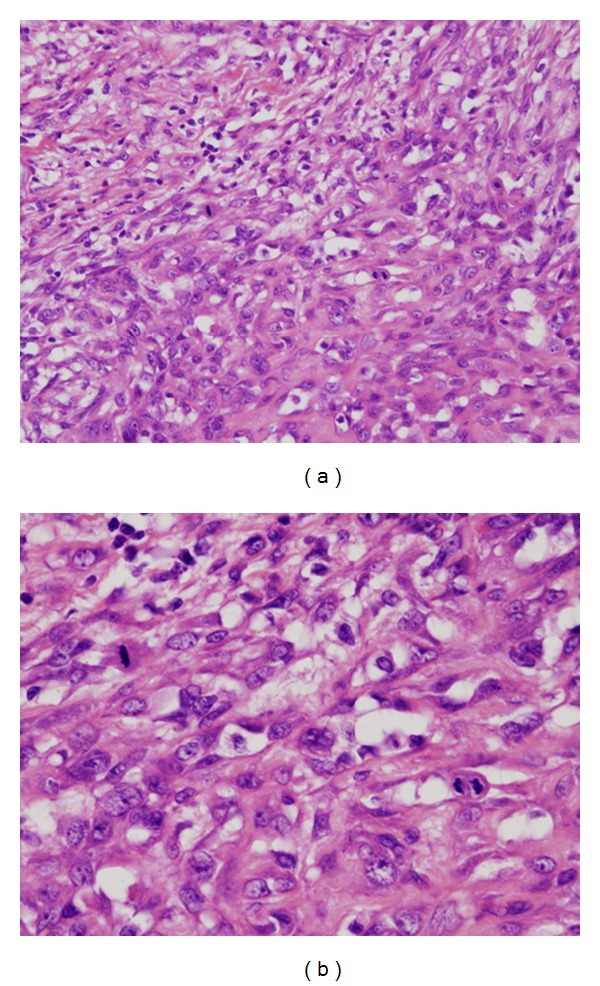
(a) Microscopic examination of primary breast tumor showing invasive proliferation of spindle-shaped and small rhombus cells and showed alveolar formations in a part of the tumor (H&E, ×200). (b) Moderate nuclear pleomorphism, size differentiation, and mitotic figures were seen (H&E, ×400).

**Figure 2:**
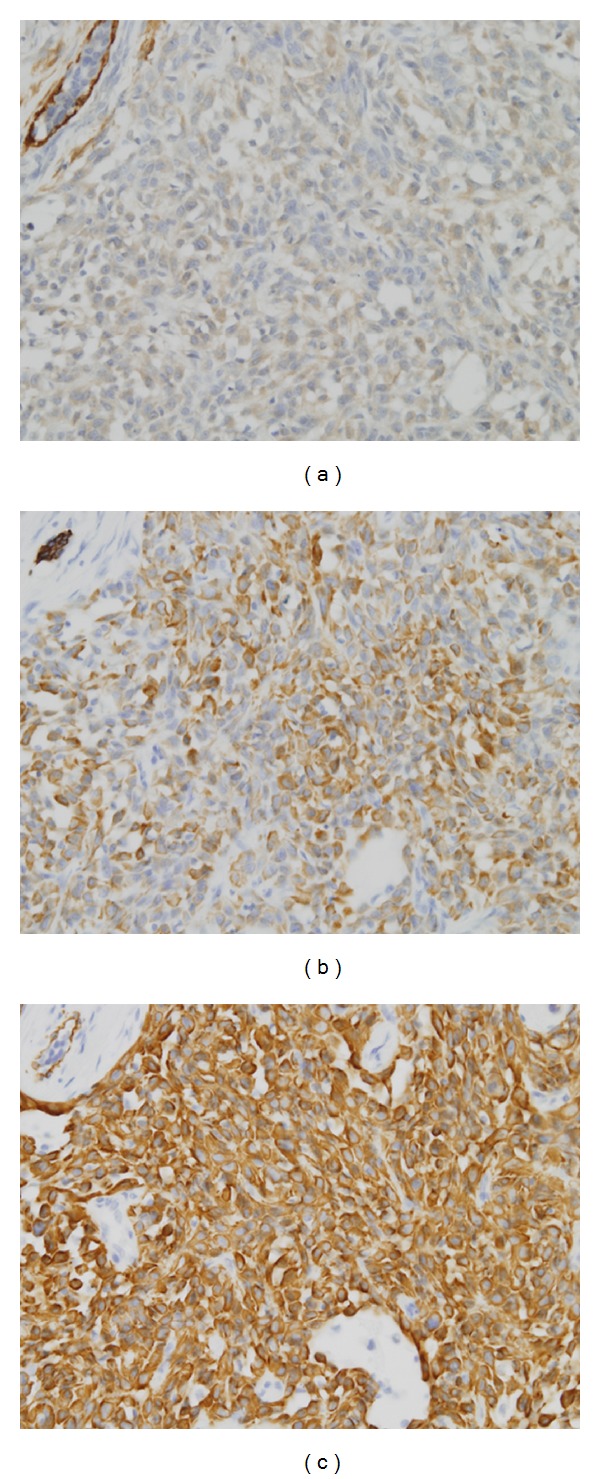
(a) Myoepithelial carcinoma revealed a diffuse cytoplasmic positivity for cytokeratin AE1/AE3 (immunohistochemical stain, ×200). (b) Cells showing calponin positivity (immunohistochemical stain, ×200). (c) Cells showing CK14 positivity (immunohistochemical stain, ×200).

Aspiration biopsy cytology was performed and breast cancer was suspected. Therefore, breast conserving surgery was performed for both diagnosis and treatment. Microscopic examination revealed an invasive proliferation of spindle-shaped and small rhombus cells and showed alveolar formations in a part of the tumor. Moderate nuclear pleomorphism and size differentiation were seen. Six mitotic figures per 10 high-power fields were present (Figure 1). Immunohistochemistry showed a diffuse cytoplasmic positivity for cytokeratin (CK) AE1/AE3, CK Cam5.2, S-100 protein, Calponin, and CK 14 (Figure 2). The cells were negative for ER, PgR, and HER2. On the basis of the histological and immunohistochemical results, this case was considered as a myoepithelial carcinoma. 

The patient received adjuvant radiotherapy. Fifty Gy was delivered to the left breast in twenty-five fractions. Fifteen months after completing treatment, a CT check of the chest was requested by the patient. CT of chest showed a left parasternal lymph node metastasis infiltrated to the sternum, although no other distant metastases were found. Treatment by further excision was decided. A histopathological examination was performed, with a review of the previous breast mass, and resulted in a histological diagnosis of metastasis.

Parasternal lymph node recurrence, pulmonary, and left kidney metastases appeared 15 months after the second surgery (30 months after initial treatment).

She received 6 cycles of a combination of carboplatin (area under the curve = 6), 200 mg/m^2^ paclitaxel, given every 3 weeks as a first line chemotherapy. After three courses, a stable disease according to RECIST criteria was obtained. The same chemotherapy was then continued for three more cycles. After six courses, CT of chest and abdomen confirmed progression of the disease. A 2nd line chemotherapy was started with combination of doxorubicin (60 mg/m^2^) and cyclophosphamide (600 mg/m^2^) (AC). The chest metastasis and her general conditions remained stable for 3 months. Six months later, all metastatic lesions except kidney had progressed and oral capecitabine (1800 mg/body) and cyclophosphamide (100 mg/body) (XC) were initiated. However, four months later, the disease showed further progression. Consequently, paclitaxel (80 mg/m^2^) and gemcitabine (1000 mg/m^2^) were started. After the third cycle, she presented with walking difficulty and was admitted to the hospital. The patient received best supportive care and died 20 months after the diagnosis of metastases.

## 3. Discussion

Myoepithelial carcinoma of the breast is extremely rare. A limited number of published reports have described myoepithelial carcinomas originating from the breast [7]. Therefore, little is known about its biological behavior. Light microscopy revealed interlacing bundles of spindle cells sometimes arranged in a storiform pattern. The cytoplasm tended to be eosinophilic or sometimes clear. An infiltrative growth pattern may be present at the periphery of the tumor [4].

Immunohistochemically, most of the antibodies used to detect myoepithelial cells and their related neoplasms are directed against keratins and myofilaments. Antibodies to selected high-molecular-weight keratins (CK5, 5/6, 14, and 17) react with most myoepithelial lesions. S100 protein is too nonspecific in reactivity to be of value in the study of these lesions. Antibodies to smooth-muscle actin, muscle-specific actin, calponin, and smooth-muscle myosin heavy chain all stain normal myoepithelial cells and most tumors containing myoepithelial cells. Given their poor degree of differentiation, myoepithelial/metaplastic carcinomas are best examined with a panel that includes all antibodies to broad-spectrum keratins, all high-molecular-weight keratins, p63, as well as antibodies to myofilaments [5]. In our case, tumors immunoreactive for low-molecular-weight keratin (CK Cam5.2), high-molecular-weight keratin (CK 14), broad-spectrum keratins (CK AE1/AE3), S100 protein, and calponin confirmed the diagnosis of myoepithelial carcinoma. Benign adenomyoepithelial lesions variably express hormone receptors in the epithelial component. However, myoepithelial carcinomas typically are completely negative for hormone receptors [5]. Our case was also negative for ER and PgR.

Myoepithelial carcinomas are treated mainly by wide surgical excision, lymph node dissection, and adjuvant radiotherapy [3]. Breast conservation surgery is an appropriate treatment in selected patients but is associated with the risk of local recurrence without adjuvant radiotherapy. The role of chemotherapy and choice of agent is not well defined [8]. To our knowledge, although a few reports referred to chemotherapies, none reported that chemotherapies were effective for recurrent myoepithelial carcinoma of the breast [6, 9]. There was only one other primary case that a metastatic myoepithelial carcinoma of the vulva had lung metastases and was successfully treated by chemotherapy [3]. 

In our recurrent case, the first treatment was by further wide excision. However, parasternal lymph node recurrence, pulmonary, and left kidney metastases appeared. We were unable to find any effective chemotherapy information to treat metastatic myoepithelial carcinoma. A chemotherapy regimen was chosen with broad activity that is used frequently for treatment of carcinoma of unknown primary origin. The patient received 6 cycles of a combination of carboplatin (area under the curve = 6), 200 mg/m^2^ paclitaxel, given every 3 Weeks. This regimen was used previously for only one case who achieved a durable pathological complete response to chemotherapy too [3]. Our case remained as a stable disease for 3 months. After that, we chose chemotherapies in accordance with primary breast cancer. However, none of those regimens (AC, XC, paclitaxel, and gemcitabine) were effective.

A few case reports have described breast myoepithelial carcinomas accompanied by distant metastasis, and although chemotherapy was mentioned, no details were provided regarding drug regimens or clinical outcomes [10, 11]. The case described here developed locoregional and distant metastases, inasmuch as many chemotherapy regimens were applied. A multidisciplinary approach is recommended for the treatment of this rare disease.
